# Palestine 100 years ago, Hans Christian Anderson’s tale of ‘The Emperor’s New Clothes’, malaria elimination and today’s failure of engagement with the community

**Published:** 2022-02-17

**Authors:** Anton Alexander

**Affiliations:** 1 BC Business Centrum, Elscot House, Arcadia Avenue, London N3 2JU, United Kingdom.

## Abstract

A century ago, Dr. I. Kligler, a Jewish Zionist in Palestine, initiated the first start anywhere in the world of a successful national malaria elimination campaign. It is little realised today that Palestine back then was drenched in malaria, rendering it uninhabitable in many areas, leaving it a country almost empty. Kligler recognised in his quest for malaria elimination firstly the need to demonstrate that malaria elimination was in fact possible. Secondly, he noted that the old colonial attitudes which then prevailed around the world towards subject populations also existed in Palestine. He realised that to be effective in educating anyone to assist with malaria elimination in such a colonial environment, that person would need to be educated, and such education had to be conducted with ‘dignity and respect’. Such an attitude is something that was (and still is in many places) sadly missing within former colonies. Kligler knew the Zionist dream of a Jewish homeland in Palestine would in all probability be an impossibility unless malaria could be defeated there. He accordingly set out to initially demonstrate on a very small scale what could be achieved by way of malaria control. With dignity and respect, he engaged with all the inhabitants, both Arabs and Jews, sometimes even individually if necessary, to educate about the disease and to explain why his anti-malarial works were necessary, thereby enlisting the inhabitants effective assistance in these works. The result was enthusiastic co-operation by the inhabitants for over twenty years which was eventually rewarded with malaria elimination. Unfortunately, the example shown by Kligler is little known to the rest of the world today, and unless communities suffering from the disease learn from such examples, the task of malaria elimination elsewhere around the world will continue to be fraught with great difficulty, and more than likely to result in failure.

## Introduction

This paper is long overdue. After the defeat by the British Army of the Turkish Army in 1918, in the final year of World War I, Palestine was administered by the British Mandate, in effect a colony-like structure. It is little appreciated today that Palestine was then thinly populated or even uninhabitable in many areas. Indeed, Palestine was then almost empty. It is also usually not appreciated that if malaria had not been eliminated in Palestine, it is doubtful the State of Israel could ever have come into existence. An example of the tangible success of the Palestine malaria elimination can be seen from the following statistic. In 1922, the British Mandate conducted a Census in Palestine [1] disclosing a total population of 757,182, including all the military. Over the following one hundred years, the area underwent a huge transformation, with the population in 2020 for the same area [2] increasing to approximately 13.5 million – an increase of almost 18 times the 1922 level.

The following brief extract from a previous paper [3] may assist in appreciating the severity of the malaria that existed in Palestine 100 years ago.

*In 1919, Dr. Manson-Bahr, a future director of the London School of Hygiene and Tropical Medicine, described Palestine as one of the most highly malarious countries in the world. He knew the conditions in Palestine as in 1918, in the final months of WWI, whilst an officer in the Royal Army Medical Corps with the British Army in Palestine, he had witnessed a force of 40,500 men lose 20,427 men in 9 weeks due to malaria. Of the 100,000 Turkish prisoners-of-war taken after their defeat in 1918 by the British Army in Palestine, 20 per cent had to be hospitalised immediately, suffering from malaria.*

It is said that a picture is worth a thousand words. The author considered that the Hans Christian Anderson’s tale of ‘The Emperor’s New Clothes’ creates an impression which can serve as a picture for the purpose of this paper. For those unfamiliar with the tale, the plot (as told in Wikipedia) is as follows.

*Two swindlers arrive at the capital city of an emperor who spends lavishly on clothing at the expense of state matters. Posing as weavers, they offer to supply him with magnificent clothes that are invisible to those who are stupid or incompetent. The emperor hires them, and they set up looms and go to work. A succession of officials, and then the emperor himself, visit them to check their progress. Each sees that the looms are empty but pretends otherwise to avoid being thought a fool. Finally, the weavers report that the emperor's suit is finished. They mime dressing him and he sets off in a procession before the whole city. The townsfolk uncomfortably go along with the pretense, not wanting to appear inept or stupid, until a child blurts out that the emperor is wearing nothing at all. The people then realize that everyone has been fooled. Although startled, the emperor continues the procession, walking more proudly than ever [4].*

For many years, historical narratives have been promoted providing a hostile account of the creation of the State of Israel in 1948 from out of Palestine. These narratives have often assumed the form of the Emperor’s New Clothes, misleadingly omitting reference to the malaria which devastated the country. Such narratives for years have thereby provided an incorrect impression that malaria in Palestine 100 years ago did not exist. In effect, it may have been an attempt to make the disease invisible!

The world has been done a great disservice by the failure before now to declare *‘the emperor is wearing nothing at all’*, to call out that Palestine 100 years ago was drenched in malaria, that it was accordingly uninhabitable in many areas. Palestine, in fact, had become desolate and neglected in many areas, and was then almost empty of inhabitants. The method and approach begun by the Zionists in 1922 to eliminate malaria in Palestine were successful, there was much to learn from the method, and the lessons from that malaria elimination are still relevant around the world and could still be applied today.

Due to the omission of reference to malaria in these misleading narratives, today’s malaria-community is likely to be unaware of the steps taken in the successful malaria elimination in Palestine all those years ago and which experience could be saving lives today. Sadly, it is likely such misleading narratives by these malaria omissions will have done harm, costing many lives over the years throughout the world today wherever malaria has existed.

### The harm done by ‘the emperor’s new clothes’

The architect of the malaria elimination campaign, Dr. I. Kligler, was a brilliant Public Health Scientist, a Jewish Zionist, who had arrived in 1920 to settle in Palestine. He was to be continuously stressing that the education of *all* the inhabitants was as important as the anti-malaria work itself. The basis of his method was destruction of *all* mosquito breeding sites to prevent or control malaria in that locality. This feat was to be achieved through continuous, thorough and systematic larval source management. Through effective education, Kligler ensured those particular breeding sites remained unproductive for mosquitoes for years on end.

For those aware of Kligler’s emphasis on education, they would have become familiar with a benchmark, a standard of education, that ensured the malaria elimination would be successful.

It is important to mention that Kligler’s malaria control and elimination were treated as a priority. Without such attention, Palestine would have remained as it had been for centuries.

It is also important to mention Kligler’s successful approach to malaria elimination differed from most of today’s methods of dealing with malaria.

Kligler had treated malaria as a local problem, and usually had used entomological skills with precision to identify and then destroy mosquito breeding sites in a particular locality. Due to the education and resulting cooperation, the local inhabitants over the years would then have maintained the anti-malaria works to ensure the breeding sites remained destroyed. Whilst Kligler’s method was dependent upon local collective assistance maintaining local anti-malaria works, each inhabitant would have still received individual education and all inhabitants would have appreciated why the maintenance was necessary. A further point to remember, all maintenance would have been visible for inspection afterwards to ensure it was up to a required standard.

Today’s methods, however, are usually not locally based and instead have tended to be more in the nature of attempts at malaria control through use of insecticide-treated nets and indoor residual-spraying, but these methods in recent years have met with disappointing progress which has slowed and stalled [5]. The method is usually dependent upon each individual inhabitant using a net and accepting their houses to be sprayed, and any inspector is completely reliant on an account provided by each inhabitant as to how a net was used the previous night.

Monitoring the success or progress of attempts at malaria-elimination is of the utmost importance, and without a reliable and accurate monitoring procedure, it is almost akin to 'flying blind’. Taking to the air with a blind pilot and without any wireless, controls or instruments can be likened to a suicide flight. The success or progress of Kligler’s method was always going to be relatively straightforward to monitor as it contained two advantages over today’s malaria methods. Firstly, the malaria works were visible, and could be physically inspected and checked to ascertain if they were adequate or had been properly maintained. Secondly, the location of the source of new cases could be more readily identified because the Kligler method always treated malaria as a local problem which could be traced back and was dealt with at a local level.

Monitoring today’s malaria-elimination methods, on the other hand, appears to be a more difficult task and the approach feels incomplete or only partially effective. Today’s malaria methods usually are applied over extensive areas including differing backgrounds, localities and situations. Such areas therefore include many variations all grouped together. Most national malaria control programmes in these areas today employ verifiable indicators which monitor malaria prevalence over time or monitor the incidence of new cases per time period. It is likely such indicators will also need to display data showing how use of insecticide-treated nets over the whole monitored area has interacted with the malaria prevalence. If it is decided a revealed trend is unsatisfactory, the difficulty with today’s malaria method is identifying ‘how, what or where is the problem’. It is then that a solution is sought for correcting or modifying the unsatisfactory trend. There are many aspects at that stage which could be affecting the trend, but finding that ‘how, what or where is the problem’ in the first place can be very difficult. It is often easily forgotten or overlooked that part of the data upon which the unsatisfactory trend has been based may not have been monitored or verified independently and which data may have been based on an oral account of the inhabitants that may, or may not be reliable. If the involved inhabitants had have been educated to a standard that Kligler would have thought acceptable, this latter point, namely the reliability of the oral account, would not have been so questionable. The inhabitants would then have had a greater appreciation and understanding of the proper use of the nets, and of the significance and importance of the monitoring.

The following will further demonstrate the harm done from the malaria community being unaware of Kligler’s experience and his teachings. Below is an extract from the only textbook he wrote in 1930 on malaria control:

*“The actual success of any control measure depends on the quality, training, and dependability of the local inspector. The selection and training of this class of personnel is, therefore, of the utmost importance in malaria control. The man should be sufficiently intelligent to grasp the elements of the theoretical basis of the work and become interested in the work as such, and to understand the responsibility resting on him. He should also be ready to do the work, and not merely stand by and order someone else to do it. These are considered essential qualities, and only such men were selected who possessed both of them.*

*The education of the inhabitants was the third, and by no means the least important element which conditioned the success of the work. Without active co-operation on the part of the people, the work would have been only partially successful. It was possible to obtain their active co-operation only after they understood fully the significance and value of the work.*

*The fourth important step in the effective organisation of a control area was the constant supervision. The work of the inspector as well as that of the physician, had to be checked at frequent intervals, particularly during the early stages. This also had an educational value because it kept the question alive, offered opportunity for advice and discussion, and continued the training. It also often enabled one to correct mistakes before the consequences could become serious, and to detect any delinquency in the work. After a man was trained and tried, he could be left a good deal on his own responsibility, but supervision and direction were always necessary” [10].*

Kligler’s comment that all measures had to be checked was common-sense, but for Kligler to remind of such a thing demonstrated its importance. Unfortunately, with regard to today’s method, such checking isn’t possible except by reliance on an oral account which may or may not be accurate.

In order to progress, to move forward with confidence, in any scientific endeavour, there must be reliable and accurate data available for decisions to be made and for solutions to be planned. Such physical evidence is unavailable with today’s malaria methods, and the reliability of whatever data is received or collected can only be treated with confidence to the extent of the apparent reliability of the witness.

A totally reliable and accurate measurement of the effectiveness of today’s malaria methods does not therefore seem to have been achieved.

### Engagement with the community

A very relevant paper published in 2021 entitled ‘Unlocking the human factor to increase effectiveness and sustainability of malaria vector control’ [6] summarised the present failings and shortcomings of today’s methods. It is not proposed to list or schedule these failings and weaknesses contained in the paper but it helpfully concludes:

*“Malaria outcomes and the success of interventions depend on access, acceptance, and consistent use of prevention measures. No matter how efficacious, a tool will remain ineffective if communities do not engage with it or use it regularly.”*

The conclusion of the article emphasised the necessity for community engagement and which is consistent with comments made at two Ifakara Health Institute Master Classes given also in 2021 by Professors Christian Lengeler [7] and Marcel Tanner [8], both acclaimed and highly experienced scientists dealing with malaria.

Professor Lengeler of the Swiss Tropical and Public Health Institute in Basel, Switzerland concluded a Master Class in June 2021 on the topic of mosquito bednets with the comment that 20% of solutions to malaria elimination involved technology, knowledge and science, and that the remaining 80% of solutions involved ‘Improved management and better engagement with the Community’.

Professor Tanner was Director of the Swiss Tropical and Public Health Institute from 1997 to 2015 and is now President of the Swiss Academy of Sciences. During a Masterclass in August 2021, he was asked for his view on the 20:80 solution, and he commented that 80% was the correct weight in his opinion. He added that the magic bullet on its own was harmless, but that the bullet became efficacious only when used with the 80% magic gun of a health and social system to bring it to, and for it to be accepted by, the people.

The scientific opinion has therefore recognised that 80%, indeed the major part, of the malaria-elimination solution is bound up with engagement with the community.

It must be understood that ‘engagement’ in this context implies a genuine meeting of the minds of the parties, not merely a passing fleeting physical contact, otherwise attempts at elimination are very often pointless [3].

It seems that whilst the expression ‘engagement with the community’ is freely referred to, there currently seems little available to realistically explain or guide how that ‘engagement’ was to be achieved. Exposure to Kligler’s Palestine experience would have assisted.

A brief study of current malaria booklets and pamphlets of instruction for those attempting engagement with the community today appear to often provide only a general theoretical well-meaning but unrealistic philanthropy. Examples [11] of such instructions seen by the author vary from theoretically-good to the weak. They sometimes are too vague and general, and without real effective checks on thoroughness of implementation. One hundred years ago, the Palestine approach was to ensure anti-malaria activities were thorough, continuous and systematic. To achieve this, the Palestine instructors explained the disease and how it was contracted, and the instructors would not move on until they felt that each inhabitant had understood the topic. If it was felt an inhabitant had not understood, an instructor would return and explain again. This may appear to be a slow process, but the anti-malaria method had to be thorough in its application, and it had to be continuous. An inhabitant who didn’t understand why this was necessary represented a gap in the anti-malaria defences [3].

But before such instruction or education could take place, it was necessary firstly to interest the inhabitants in malaria control or elimination, to cause the inhabitants to realise that a death from malaria was not just a fact of life. Instead, the inhabitants had to realise that such a death was a tragedy. The inhabitants had to believe malaria was not inevitable, therefore fatalism had to be overcome. The commencement of the successful Zionist malaria elimination in Palestine 100 years ago was a demonstration of an effective engagement with the community. Palestine was one of the first places to throw off some of the world’s old colonial attitudes which it did by engaging with dignity and respect all the inhabitants (both Arabs and Jews). This resulted in an extraordinarily strong and resilient cooperation by the inhabitants, Jews and Arabs, in the necessary anti-malaria works, lasting for years and years, and which cooperation was to rid the country of the disease [3].

I ponder the point and ask the question to the malaria-community: Are inhabitants today truly treated with respect and dignity? Is the approach and engagement with inhabitants the same that each one in the malaria community would honestly want for themselves? Is there a whiff of old-style patronage about the malaria community’s approach?

Palestine, the size of Wales, 100 years ago was almost empty, and Kligler did not have to deal with populations on the same scale as that encountered today by many of us. At a lecture in 1917, given at the Royal Geographical Society, Palestine was described: “The land is by no means uninhabited, and in its desolated condition it will scarcely support its reduced population” [9]. But this also reduced the pressure and enabled Kligler to reflect on each step, to test the principle that unless a population cooperates and assists with the malaria elimination works, such works are likely to be of little avail.

Incidentally, Kligler wrote of the difficulty of understanding at first sight how a country as sparsely settled as Palestine could have such a disease so widely spread and epidemics so continuous. He wrote that epidemics are usually correlated with crowding. He continued that the answer was furnished by a study of the social and economic conditions prevailing there, and that because the country was small and undeveloped, there was a constant active movement of the various population groups including the Bedouin and those on annual pilgrimages. He noted that this movement was as effective in spreading malaria and maintaining its epidemicity as it would be in the case of any other infectious disease [10].

## Conclusions

This paper is not intended as a panacea for the world’s malaria problems. Each country, each district is different from the next and has to find its own individual method to deal with malaria for that location. The intention of this paper is to demonstrate that whilst there are many problems with today’s methods, this paper only wishes to draw attention to the overlooked lessons that can be learnt from Kligler’s successful malaria elimination.

Kligler would probably have been dismayed by the inhabitants’ apparent general lack of co-operation and assistance today in many countries and areas, a cooperation and assistance with which he had become accustomed in Palestine as the norm. He would also in all probability have been disappointed by an apparently insufficient emphasis on a benchmark or standard of education which he had applied throughout his Palestine experience.

Unless the inhabitants today properly understand the problem and also the reasons for the instructions that they are given concerning any malaria activity, they will seek to avoid anything that is tedious, repetitive or arduous. More resources for education, not more nets, should now be found, and much more time must be allowed for such personal education. The malaria community must realise that it is greatly dependent upon the co-operation and assistance, and without which any malaria elimination exercise may be pointless.

Further, through education, if inhabitants understood the disease, and appreciated the necessity for conducting anti-malaria activities, the monitoring, accuracy and reliability of the data collected afterwards is likely to greatly improve.

Therefore, in response to today’s weary resignation of repeating more and more of the same activity, of sometimes doubtful value, again and again, why not instead consider and apply Kligler’s stress and emphasis on education which today can provide a greater control leading to eventual elimination of the disease. Be patient and be thorough. And other than dealing with education, concentrate anti-malaria activities only in those areas where education has already been successfully applied and where there is, therefore, already an appreciation and understanding of what is trying to be achieved.

If you will it, it is no dream.

## References

[1] https://archive.org/details/PalestineCensus1922.

[2] https://data.worldbank.org/country.

[3] Alexander A (2021). What underscored successful malaria elimination in Palestine 100 years ago? Effective Education.. MalariaWorld J..

[4] https://en.wikipedia.org/wiki/The_Emperor%27s_New_Clothes.

[5] (2021).

[6] Monroe A (2021). Unlocking the human factor to increase effectiveness and sustainability of malaria vector control.. Malar. J..

[7] https://www.youtube.com/watch?v=Y6fA8ViX8c8&t=8551s.

[8] https://www.youtube.com/watch?v=s28PB_74dKo.

[9] Masterman EWG (1917). Palestine: Its Resources and Suitability for Colonization.. Geogr. J..

[10] Kligler IJ (1930). The epidemiology and control of malaria in Palestine..

[11] (2020).

